# Identification of Psychological Profiles of Cancer Patients Undergoing Fertility Preservation Counseling

**DOI:** 10.3390/jcm12124011

**Published:** 2023-06-12

**Authors:** Valentina Elisabetta Di Mattei, Paola Taranto, Gaia Perego, Serena Desimone, Paola Maria Vittoria Rancoita, Antonio Catarinella, Raffaella Cioffi, Giorgia Mangili, Valeria Stella Vanni, Massimo Candiani

**Affiliations:** 1 School of Psychology, Vita-Salute San Raffaele University, 20132 Milan, Italy; dimattei.valentina@hsr.it (V.E.D.M.); a.catarinella@studenti.unisr.it (A.C.); 2 Clinical and Health Psychology Unit, IRCCS San Raffaele Scientific Institute, 20132 Milan, Italy; s.desimone2@studenti.unisr.it; 3 University Centre for Statistics in Biomedical Sciences (CUSSB), Vita-Salute San Raffaele University, 20132 Milan, Italy; rancoita.paolamaria@unisr.it; 4 School of Medicine, Vita-Salute San Raffaele University, 20132 Milan, Italy; candiani.massimo@hsr.it; 5 Obstetrics and Gynecology Unit, IRCCS San Raffaele Scientific Institute, 20132 Milan, Italy; cioffi.raffaella@hsr.it (R.C.); mangili.giorgia@hsr.it (G.M.); vanni.valeria@hsr.it (V.S.V.)

**Keywords:** oncofertility, oocyte cryopreservation, psycho-oncology, cancer, fertility, reproductive health, quality of life

## Abstract

Gonadotoxicity is one of the most distressing side effects of cancer treatment. Fertility preservation strategies should be included during the treatment pathway to prevent the risk of infertility, but the decision to preserve fertility often represents a challenging process that carries an emotional decision-making burden. The aim of this study is to characterize the psychological profiles of women undergoing fertility preservation counseling and to better understand their features. Eighty-two female cancer patients were included in the study. They were asked to complete a battery of self-administered tests which evaluated socio-demographic characteristics, defense mechanisms, depression, anxiety, and representations regarding the importance of parenthood. Based on the psychometric variables, cluster analysis identified four groups which showed significantly different combinations of these psychological characteristics. An additional analysis was performed to evaluate if sociodemographic variables were associated with the four groups, but the results did not show significant differences. These results suggest that very diverse psychological profiles may lead cancer patients to attend oncofertility counseling and choose fertility preservation. For this reason, all patients in childbearing age should have the opportunity to receive appropriate fertility preservation counseling in order to make an informed decision that could have an important impact on their long-term quality of life.

## 1. Introduction

Advances in cancer diagnosis and treatment fostered an increase in recovery and survival rates. Consequently, focusing on the long-term effects and quality of life after the disease is now essential.

In particular, among the side effects, women consider gonadotoxicity to be one of the most distressing late effects of cancer treatment [1]. Indeed, treatments can compromise ovarian reserve, hormonal balance, or the functioning of reproductive organs [2], inducing a high risk of temporary or permanent infertility. In order to prevent such risks, the American Society of Clinical Oncology recommends fertility preservation methods such as cryopreservation of embryos, oocytes, or ovarian tissue, and it has also updated its guidelines to include ovarian suppression in the treatment pathway [2]. Fertility preservation strategies, however, may add an additional emotional burden to women who already need to experience an initial diagnosis and make decisions about treatments. In fact, the decision to undertake fertility preservation, which generally takes a few weeks, may result in the delay of cancer treatment and is often made without receiving adequate counseling. Because of these difficulties, it is not surprising that only half of patients decide to undergo fertility preservation [3].

However, infertility can lead to negative consequences for individuals’ and couples’ long-term psychological well-being and quality of life [4,5,6]. Therefore, it is important that patients receive adequate oncofertility counseling in order to make a conscious and informed choice based on their own goals and values.

The Oncofertility Unit at the San Raffaele Scientific Institute is an Italian reference center for fertility preservation in oncology; therefore, patients are referred there both from within other departments in the hospital as well as from other Italian hospitals. The aim of this study is to try to characterize the psychological profiles of women undergoing fertility preservation counseling in order to better understand their features. The psychometric variables assessed in this study were selected based on what the literature indicates to be relevant and/or predictive for the choice to pursue fertility preservation: defense mechanisms, anxiety and depression, and the importance of parenthood [6,7,8,9]. In this study, psychological profiles are identified by analyzing psychometric variables with multivariate statistical techniques that consider their mutual interactions.

## 2. Materials and Methods

### 2.1. Data Source

Female patients referred to the Oncofertility Unit of the IRCCS San Raffaele Scientific Institute after cancer diagnosis and before gonadotoxic treatment between March 2016 and May 2022 were invited to participate in the study. This study was carried out following the guidelines of the San Raffaele Hospital Ethics Committee (protocol N. 149/INT/2019) and in accordance with the Declaration of Helsinki.

Eligibility criteria were being at least 18 years old, having recently received a diagnosis of cancer, speaking and understanding Italian, having at least an elementary school degree, not refusing to preserve fertility, and agreeing to voluntarily participate in the research through written informed consent.

The patients were invited to participate in the research during a counseling session with a psychologist, before their first oncofertility appointment. The psychologist explained the objectives and methods of the research and obtained the patients’ informed consent to participate. The final sample consisted of 82 patients.

### 2.2. Measures

The participants were asked to complete a battery of self-report questionnaires.

An ad hoc socio-demographic questionnaire was used to collect socio-demographic characteristics such as age, education, relationship status, number of children, and previous miscarriages.

The Response Evaluation Measure-71 (REM-71) [10] measures defense mechanisms in adults and adolescents. It consists of 71 items, with each item scored on a nine-point Likert scale from “strongly disagree” to “strongly agree”.

The REM-71 considers twenty-one defense mechanisms, and the score of each defense mechanism is given by the average of the scores of the items describing that defense.

Factorial analysis allowed for the identification of two factors based on the level of maturity of these defense mechanisms. Factor 1 (F1) expresses the global score regarding the immature defense mechanisms that can distort reality and most commonly lead to less adaptive functioning. This cluster is divided into fourteen defenses: acting out, splitting, displacement, fantasy, omnipotence, dissociation, projection, repression, undoing, withdrawal, somatization, passive aggression, conversion, and sublimation.

Factor 2 (F2) represents the global score of mature defense mechanisms, which mitigate an unwelcome reality and allow for more adaptive functioning. This cluster consists of seven defense mechanisms: altruism, idealization, denial, intellectualization, humor, reactive formation, and suppression.

Higher values in both factors correspond to a greater use of these defense mechanisms.

The overall Cronbach’s alpha values for the two factors are 0.84 for F1 and 0.69 for F2 [10].

The Italian version of the questionnaire was used in this study. This version has an internal consistency of 0.88 for F1 and 0.73 for F2 [11]. Test–retest reliability ranges from 0.93 for F1 to 0.95 for F2 [12]. Prunas et al. [13] identified a score of 4.40 as the clinical cut-off only for F1.

The Beck Depression Inventory (BDI-II) [14] detects the severity of depression in adults and youth over 13 years of age. It contains 21 items to investigate cognitive, affective, and somatic symptoms of depression, with each item scored on a four-point Likert scale from 0 to 3, depending on the severity of the content. Individual item scores are added up to create a total score ranging from 0 to 63. Different severity levels have been defined on an empirical basis [15]: minimum depression (scores of 0 to 13), mild depression (scores of 14 to 19), moderate depression (scores of 20 to 28), and severe depression (scores of 29 to 63).

The BDI-II has excellent psychometric properties: Cronbach’s alpha coefficient ranges from 0.92 for outpatient samples to 0.93 for nonclinical samples, and test–retest reliability is greater than 0.90 [14].

The Italian version of the questionnaire was used in this study [16]. This version shows a good internal consistency, with Cronbach’s alpha coefficient between 0.80 and 0.86 and test–retest reliability of 0.76 [16].

The State–Trait Anxiety Inventory (STAI-Y) [17] assesses anxiety symptoms, differentiated into state and trait anxiety. It consists of 40 total items that are answered using a four-point Likert scale from 1 to 4, depending on the content. The total score is given by the sum of the individual items and can range from 20 to 80. Scores are grouped into three categories [18]: low anxiety (scores of 20 to 39), medium anxiety (scores of 40 to 59), and high anxiety (scores of 60 to 80).

The STAI-Y Cronbach’s alpha coefficient ranges from 0.83 to 0.92 for state scores and 0.86 to 0.92 for trait scores [19]. The state test–retest reliability is 0.40 while the trait test–retest reliability is 0.86 [20].

The Italian version of the questionnaire was used in this study [21]. The internal consistency values range between 0.91 and 0.95 for the State anxiety scale (depending on the sample) and between 0.85 and 0.90 for Trait anxiety scale. The test–retest reliability is 0.49 for state subscale and 0.82 for trait anxiety [21].

The Fertility Problem Inventory (FPI) [22] measures infertility-related stress. Infertility is associated with a form of chronic stress that can bring psychological difficulties; however, the experience of stress is the product of a combination of several factors. For this reason, the FPI is a multi-domain measurement instrument. Specifically, the questionnaire consists of 46 items organized into five subscales defined as “Social concern”, “Sexual concern”, “Relational concern”, “Need for parenthood”, and “Rejection of a child-free lifestyle”, with answers given on a 6-point Likert scale from “strongly disagree” to “strongly agree”. The global stress measure is given by summing the scores of all five scales.

All FPI scales show good reliability with Cronbach’s alpha values between 0.77 and 0.93 [22].

The Italian validation study [23] of the questionnaire showed that the scales defined in the original version have adequate internal consistency, with alpha reliability coefficient values between 0.71 and 0.93 for all subscales, excluding the “Rejection of a child-free lifestyle” subscale (α = 0.66). Moreover, both Moura-Ramos et al. [24] and Donarelli et al. [23] found a strong correlation (r = 0.55 and r = 0.64, respectively) between the two subscales “Need for parenthood” and “Rejection of a child-free lifestyle”. In particular, Moura-Ramos et al. [24] proposed a bifactorial model of FPI: the first domain “Impact on Life Domains” includes the areas of life affected by the experience of infertility (i.e., the subscales “Social concern”, “Sexual concern”, and “Relational concern”); the second domain “Representations about the Importance of Parenthood” includes beliefs about parenthood and the presence of children in couples lives (i.e., the subscales “Need for parenthood” and “Rejection of child-free lifestyle”) [24].

In this study, the Italian version of the FPI was administered integrally [23], but we only considered the construct “Representations about the importance of Parenthood” (as defined in [24]) in our analysis because it has been shown previously to predict high motivation to undergo fertility preservation techniques [25]. For the sake of conciseness, we refer to this construct as “Importance of parenthood”.

### 2.3. Statistical Analysis

Numerical variables were summarized with median and interquartile range (IQR), while categorical variables were described in terms of absolute and relative frequencies.

A hierarchical cluster analysis was used to identify groups that were internally homogeneous and distinct from the others with regard to the psychometric variables. Considering the nature of the scales, Euclidean squared distance was used as distance and Ward’s method of clustering was used. For the selection of the number of groups, the dendrogram and agglomeration schedule were analyzed, and the relative increment was calculated. The optimal solutions had two or four groups. In the final evaluation, the four groups solution was chosen, because it optimized both the criterion of internal homogeneity and external heterogeneity and the interpretation of the groups themselves. The derivation of profiles describing the four groups was performed by comparing the distributions of psychometric variables among the groups. This analysis was conducted with the Kruskal–Wallis test, a post hoc analysis with Dunn’s test, and *p*-values adjusted with Bonferroni’s correction.

Subsequently, the Kruskal–Wallis test for numerical variables and Fisher’s test for categorical variables were used to compare sociodemographic variables among the four groups.

The level of significance was set at 0.05. All statistical analyses were carried out with IBM SPSS Statistics version 27 statistical software.

## 3. Results

Descriptive statistics for sociodemographic variables on the total sample are reported in Table 1. The sample consisted of 82 female cancer patients, aged between 20 and 42 years old (median = 33; IQR = 28.75–37.00). More than half of the sample (64.6%) had at least a bachelor’s degree, and most of them were in a romantic relationship (80.5%). Only 15.9% already had children, and 18.3% had one or more previous miscarriages.

Table 2 shows the descriptive statistics for the six psychometric variables. Factor 1 of the REM-71, which refers to the global score of immature defense mechanisms, is lower (median = 3.92; IQR = 3.05–4.48) than the clinical cut-off of 4.40 identified by Prunas et al. [13]. Values for Factor 2 of the REM-71, representing the global score of mature defense mechanisms, fall into the upper part of the range of possible values (median = 5.94; IQR = 5.55–6.44; range = 1–9). Depression in the sample displays a distribution (median = 10; IQR = 6.00–15.00) that indicates a minimum level of depression (range = 0–13). The distribution of state anxiety (median = 45; IQR = 38.00–56.25) indicates that patients have a medium level of anxiety (score between 40 and 59), while the distribution of trait anxiety (median = 39; IQR = 32.00–45.00) falls into the upper limit of the range of low anxiety (score between 20 and 39). The scores of the FPI construct “Importance of parenthood” (median = 64; IQR = 49.00–77.00) suggest that the sample reports medium levels in the range of possible values (range = 18–108).

A cluster analysis conducted on the sample identified four groups based on the psychometric variables.

Descriptive statistics and the comparisons of the distributions of psychometric variables among the four groups are reported in Table 3 (with the corresponding post hoc analysis in Table 4). These results show that the groups are significantly different from each other in all the scales.

From the post hoc analysis of Factor 1 of the REM-71, group 1 is characterized by values that are significantly different from the other groups (group comparison: 1 vs. 2 *p* = 0.002; 1 vs. 3 *p* < 0.001; 1 vs. 4 *p* = 0.004). Specifically, in this group the values for Factor 1 are lower than all the other groups (median = 2.76 vs. 3.91 for group 2, 4.10 group 3 and 4.40 group 4), implying that women in this group use immature defense mechanisms to a lesser extent.

Regarding the REM-71 Factor 2, group 2 is significantly different than the other groups (group comparison: 4 vs. 2 *p* = 0.004; 3 vs. 2 *p* = 0.004; 1 vs. 2 *p* = 0.024), with values that are higher compared to the other groups (median = 6.55 vs. 5.81 for group 1, 5.85 for group 3, 5.55 for group 4). This supports that patients in this group use mature defense mechanisms to a greater extent.

For the BDI-II, group 1 shows significantly lower scores than groups 3 and 4 (median = 6.00 vs. 17.00 and *p* < 0.001 for group 3, vs. 15.00 and *p* = 0.003 for group 4) and group 3 shows higher scores than group 2 (median = 17.00 vs. 9.00, *p* < 0.001). Specifically, patients in group 1 have lower levels of depression, which falls within the range of minimum depression (range 0 to 13), while those in group 3 report higher levels, which falls within the range of mild depression (range 14 to 19).

Regarding state anxiety, group 3 is significantly different from groups 1 and 2 (group comparison: 1 vs. 3 *p* < 0.001; 2 vs. 3 *p* < 0.001). Scores in this group are in fact significantly higher than groups 1 and 2 (median = 60.00 vs. 36.00 for group 1, 41.00 for group 2) and fall within the range of high anxiety scores (range 60 to 80). Regarding trait anxiety, groups 1 and 2 are significantly different from groups 3 and 4 (group comparison: 1 vs. 3 *p* < 0.001; 1 vs. 4 *p* < 0.001; 2 vs. 3 *p* = 0.004; 2 vs. 4 *p* = 0.004). In particular, groups 1 and 2 show values (median group 1 = 31.00 and group 2 = 38.00) corresponding to low trait anxiety (range 20 to 39), while groups 3 and 4 have values (median group 3 = 45.00 and group 4 = 47.00) that correspond to moderate anxiety (range 40 to 59).

Finally, for the FPI domain “Importance of parenthood”, groups 4 and 1 are significantly different from groups 2 and 3 (group comparison: 4 vs. 2 *p* < 0.001; 4 vs. 3 *p* < 0.001; 1 vs. 2 *p* = 0.005; 1 vs. 3 *p* < 0.001). In particular, groups 4 and 1 show lower values (median group 4 = 46.00 and group 1 = 52.00) than groups 2 and 3 (median group 2 = 70.00 and group 3 = 75.00); thus, women in groups 4 and 1 seem to attribute less importance to parenthood than the others.

In conclusion, group 1 is characterized by low levels of immature defense mechanisms, minimum levels of depression, low levels of trait anxiety, and lower importance of parenthood. Group 2 has higher levels of mature defense mechanisms, low levels of trait anxiety, and high importance of parenthood. Group 3 shows higher levels of depression (corresponding to a mild depression), high levels of state anxiety and moderate levels of trait anxiety, and higher importance of parenthood. Finally, group 4 appears to have moderate levels of trait anxiety and lower importance of parenthood.

An analysis was conducted to characterize the groups identified by the clustering with respect to sociodemographic variables. Table 5 shows that there were no significant differences in the four groups. Slight differences, although nonsignificant, concern a lower prevalence of patients involved in a relationship in group 4 (55.6%) compared to other groups (percentage greater than or equal to 80%).

## 4. Discussion

Oncofertility is a key element within the cancer treatment path for women in childbearing age. When a woman receives a cancer diagnosis, her goals, values, and emotional experiences are redefined. In this context of great change, every woman must make crucial decisions for survival and also for the quality of life after recovery. Certainly, the preservation of fertility is part of these decisions. Current medical knowledge enables clinicians to fulfil the need for parenthood through different fertility preservation techniques, but the decision to undergo this path can be challenging from a medical and psychological standpoint. Women with functional personality traits and a defensive style, in association with low levels of depression and trait anxiety, could have a proactive attitude and better psychological adjustment to the disease and a projection toward the future [26]. However, considering that the most important predictor of a high motivation to undergo fertility preservation is the strong desire for parenthood, it is important that, regardless of the individual characteristics, patients receive adequate oncofertility counseling in order to make a conscious and informed choice based on their own goals and values [25].

As far as we know, some studies in the literature have investigated the psychological factors associated with fertility preservation in cancer patients, but very few studies have attempted to outline the typical profiles of women accessing oncofertility counseling. The aim of our study was to characterize the typical psychological profiles of women undergoing fertility preservation counseling after a cancer diagnosis.

In this study, through cluster analysis we identified four groups characterized by different levels of mature and immature defense mechanisms, state and trait anxiety, depression, and the importance of parenthood. Our results allow for some considerations about the profiles of women accessing oncofertility counseling. The literature, although scarce regarding this topic, recurrently reports that the importance of parenthood is the most crucial factor influencing the choice to preserve fertility before cancer treatments [8,25,27]. Indeed, biological motherhood plays an important role in women’s lives, and threats to reproduction may cause significant distress. These specific concerns mediate the relationship between the importance of parenthood in women’s lives and their quality of life [28]. The interaction among the desire for parenthood and other psychological factors, therefore, could orient patients during the decision-making process.

The four groups identified in our sample show different psychological characteristics. The different features of some of our groups seem to be consistent with the few studies already available in the literature [6,7]. However, it is important to highlight that no study in the existing literature seems to define patients’ profiles by using the exact same variables considered in our study, various measures can be used to collect data, and the aims of the other studies are different; therefore, any comparison must be approached with extreme prudence.

Considering these premises, our results show that group 1 of the sample consists of women with low levels of immature defense mechanisms, minimum levels of depression, low levels of trait anxiety, and less importance of parenthood compared to the other groups. These psychological aspects are similar to those reported by patients included in the study by Ussher at al. [6]. In this study, psychological distress was assessed with the Kessler Psychological Distress Scale (K10) [29], which provides a comprehensive measure of psychological distress through items investigating anxiety and depressive symptoms. Although this study used only one scale to measure both variables—while our study uses two different tools and has a different sample size—both studies include patients who have low anxiety, minimum depression, and importance of parenthood that can be classified as low in the range of scores on the questionnaire used to investigate this construct (the FPI). The relationship among the variables could be explained by assuming that women who give relatively low importance to parenthood also have lower levels of anxiety and depression linked to the possibility of cancer-related infertility.

Group 2, on the other hand, consists of women who have higher levels of mature defense mechanisms, low levels of trait anxiety, and high importance of parenthood. These psychological characteristics are similar to the sample of women included in the study by Lawson et al. [7]. In fact, the patients in group 2 of our study and the sample of cancer patients intending to preserve fertility in the study by Lawson [7] have moderate state anxiety, low trait anxiety, no or minimum depression, and a medium to high desire for parenthood. In the view that greater importance of parenthood was directly associated with higher levels of depressive symptoms [28], Lawson et al. [7] hypothesize that the discrepant relationship between these two variables in their sample could be explained by a common tendency to underreport mental health symptoms as a function of social desirability, thus showing low depression scores but high levels of importance of parenthood.

Groups 3 and 4 of our study do not seem to be comparable with any study in the literature. In particular, group 3 includes patients with higher depression than the other groups (although mild), high state anxiety, moderate trait anxiety, and high importance of parenthood. These characteristics might suggest that this type of patients might be more focused on their mental health and, consequently, primarily oriented toward the treatment of the disease rather than future parenthood. However, an alternative argument could be that the high anxiety and depression levels, as compared to the other groups, could depend on the patient’s high investment in parenthood. Therefore, the higher levels of anxiety and depression might reflect their concerns about the risk of infertility. As such, one could hypothesize that the high need for parenthood is the factor that leads this type of patient toward oncofertility counseling.

Group 4 shows mild levels of depression, moderate levels of state and trait anxiety, and low importance of parenthood when compared to the other groups. Such features could suggest that the approach to counseling of these women could be influenced by external factors and not by an intrinsic motivation to preserve fertility. This group in particular highlights the importance of paying attention and guaranteeing counseling for all cancer patients—even women who display characteristics that are different from what one would expect from women interested in fertility preservation might apply for oncofertility counseling. However, it is important to highlight that this group has a small sample size.

In conclusion, the studies in the literature that have investigated variables associated with oncofertility counseling and preservation usually consider these patients as a single population with common features. Instead, this study highlights the existence of subgroups of patients with very different combinations of psychological characteristics, whom all seek counseling in oncofertility. Healthcare professionals should take into consideration this complexity, since the presence of characteristics which might suggest a lower interest in future parenthood should not limit the referral to Oncofertility Units, and adequate information and support should be provided to each patient in order to promote and facilitate an informed decision.

This is supported by the fact that the groups of patients identified in this study were not significantly different with respect to sociodemographic variables such as age, marital status, presence of children, previous miscarriages, and education. However, this result could be also explained by the nature of our sample, which has the limitation to be relatively small and homogeneous for the sociodemographic variables investigated.

As far as we know, the present study is one of the few that investigated the psychological characteristics of women undergoing fertility preservation counseling. In the available literature, there seems to be no other study that has specifically tried to delineate different profiles of patients accessing oncofertility counseling.

However, the sample is relatively small, and it was recruited from a single referral center. Related to this, the cluster analysis divided the sample into four groups, one of which is numerically much smaller than the others. Therefore, the representativeness and the possibility of robust comparisons among groups cannot be guaranteed.

Future studies could collect data on larger samples that are more representative of the population of cancer patients of childbearing age. It would also be desirable to recruit patients who refuse the possibility of oncofertility counseling in order to delineate typical profiles of women who decide to decline this opportunity, and the comparison between profiles of women who decide to seek or not oncofertility counseling could be useful for clinical practice. Furthermore, it could be interesting to investigate more in depth the interaction between the different factors in order to understand their influence on the decision-making process and in the characterization of patient profiles.

## 5. Conclusions

Our findings indicate that there are distinct groups of cancer patients who exhibit varying psychological traits but opt for fertility preservation. This new knowledge is clinically relevant in order to be aware of the importance of offering oncofertility counseling to all patients of childbearing age. Physicians sometimes do not suggest referral to Oncofertility Units for different reasons, including the assessment that the woman is not primarily interested in future parenthood or that she lacks the cognitive and emotional resources to take on fertility preservation techniques [30,31]. Instead, this study supports the evidence of the existence of a variety of complex psychological profiles that may lead a woman to access oncofertility counseling and opt for fertility preservation. Consequently, it would be desirable that all patients of childbearing age receive adequate counseling from a specialized multidisciplinary team, which can provide the relevant information and offer necessary supports to make an informed decision that can have an important impact on a patient’s psychological well-being and long-term quality of life. Developing multidisciplinary care plans, including educational and psychosocial interventions, is critical in oncology to foster better adherence to treatment and psychological adjustment to the disease [32].

## Figures and Tables

**Table 1:** Descriptive statistics of sociodemographic variables in the sample.

Variable	*n* (%)
Age, median [IQR]	33.00 [28.75; 37.00]
In a relationship	66 (80.5%)
Children	13 (15.9%)
Previous miscarriages	15 (18.3%)
Bachelor’s degree	53 (64.6%)

IQR = interquartile range.

**Table 2:** Descriptive statistics of psychometric variables in the sample.

Variable	Median [IQR]
REM F1	3.92 [3.05; 4.48]
REM F2	5.94 [5.55; 6.44]
BDI-II	10.00 [6.00; 15.00]
STAI-State	45.00 [38.00; 56.25]
STAI-Trait	39.00 [32.00; 45.00]
FPI importance of parenthood	64.00 [49.00; 77.00]

REM = Response Evaluation Measure; F1 = Factor 1; F2 = Factor 2; BDI = Beck Depression Inventory; STAI = State–Trait Anxiety Inventory; FPI = Fertility Problem Inventory.

**Table 3:** Comparison of psychometric scales in the four groups identified by cluster analysis.

Variable	Group 1 (*n* = 27)	Group 2 (*n* = 20)	Group 3 (*n* = 26)	Group 4 (*n* = 9)	*p*-Value
REM F1	2.76 [2.37; 3.65]	3.91 [3.69; 4.65]	4.10 [3.88; 4.71]	4.40 [3.93; 4.64]	<0.001
REM F2	5.81 [5.69; 6.40]	6.55 [5.96; 6.97]	5.85 [4.80; 6.35]	5.55 [5.08; 5.83]	0.001
BDI-II	6.00 [3.00; 8.00]	9.00 [6.25; 10.75]	17.00 [13.00; 20.50]	15.00 [9.50; 19.50]	<0.001
STAI-State	36.00 [32.00; 42.00]	41.00 [38.00; 45.00]	60.00 [55.00; 66.25]	47.00 [40.50; 53.00]	<0.001
STAI-Trait	31.00 [29.00; 35.00]	38.00 [32.50; 41.00]	45.00 [41.75; 51.25]	47.00 [44.50; 58.00]	<0.001
FPI importance of parenthood	52.00 [43.00; 75.00]	70.00 [62.25; 84.50]	75.00 [64.75; 81.75]	46.00 [25.00; 49.50]	<0.001

**Table 4:** Adjusted *p*-value of post hoc comparisons.

Variable	1 vs. 2	1 vs. 3	1 vs. 4	2 vs. 3	2 vs. 4	3 vs. 4
REM F1	0.002	<0.001	0.004	1.000	1.000	1.000
REM F2	0.024	1.000	1.000	0.004	0.004	1.000
BDI-II	0.269	<0.001	0.003	<0.001	0.003	1.000
STAI-State	1.000	<0.001	0.165	<0.001	1.000	0.067
STAI-Trait	0.096	<0.001	<0.001	0.004	0.004	1.000
FPI importance of parenthood	0.005	<0.001	0.633	1.000	<0.001	<0.001

**Table 5:** Comparison of sociodemographic variables in the four groups identified by cluster analysis.

	Group 1 (*n* = 27)	Group 2 (*n* = 20)	Group 3 (*n* = 26)	Group 4 (*n* = 9)	*p*-Value
Age, median [IQR]	31.00 [29.00; 35.00]	32.00 [26.00; 37.00]	33.50 [29.75; 38.25]	33.00 [27.50; 36.00]	0.442
In a relationship *n* (%)	22 (81.5%)	16 (80.0%)	23 (88.5%)	5 (55.6%)	0.233
Children, *n* (%)	7 (25.9%)	2 (10.0%)	4 (15.4%)	0 (0.0%)	0.283
Previous miscarriages, *n* (%)	6 (22.2%)	1 (5.0%)	7 (26.9%)	1 (11.1%)	0.241
Bachelor’s degree, *n* (%)	15 (55.6%)	11 (55.0%)	19 (73.1%)	8 (88.9%)	0.188

## Data Availability

Data cannot be shared because participants did not provide informed consent for this.
